# FOXN1 in thymus organogenesis and development

**DOI:** 10.1002/eji.201545814

**Published:** 2016-08-12

**Authors:** Harsh Jayesh Vaidya, Alberto Briones Leon, C. Clare Blackburn

**Affiliations:** ^1^ MRC Centre for Regenerative MedicineInstitute for Stem Cell ResearchSchool of Biological SciencesEdinburghUK

**Keywords:** cTECs, Forkhead, FOXN1, mTECs, Nude mice

## Abstract

Development of the primary T‐cell repertoire takes place in the thymus. The linked processes of T‐cell differentiation and T‐cell repertoire selection each depend on interactions between thymocytes and thymic stromal cells; in particular, with the epithelial cells of the cortical and medullary thymic compartments (cortical and medullary thymic epithelial cells; cTECs and mTECs, respectively). The importance of the thymic epithelial cell lineage in these processes was revealed in part through analysis of *nude* (*nu/nu*) mice, which are congenitally hairless and athymic. The *nude* phenotype results from null mutation of the forkhead transcription factor FOXN1, which has emerged as a pivotal regulator both of thymus development and homeostasis. FOXN1 has been shown to play critical roles in thymus development, function, maintenance, and even regeneration, which positions it as a master regulator of thymic epithelial cell (TEC) differentiation. In this review, we discuss current understanding of the regulation and functions of FOXN1 throughout thymus ontogeny, from the earliest stages of organogenesis through homeostasis to age‐related involution, contextualising its significance through reference to other members of the wider Forkhead family.

## Introduction

**Figure 1:**
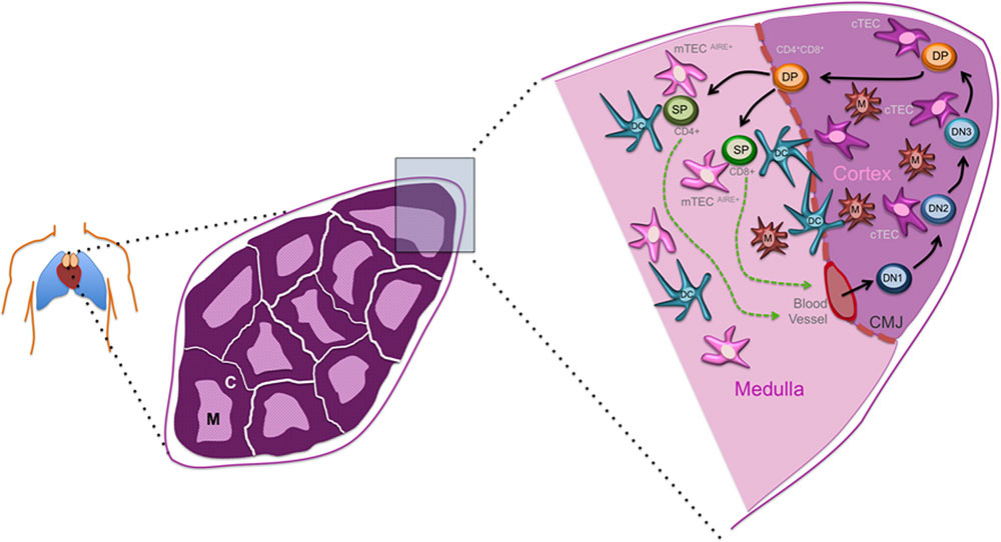
Thymus structure and development. Schematic representation of a human thymus. Left panel shows location of the thymus, at the midline above the heart. Middle panel shows representation of a section through a young thymus, indicating the thymic cortex (c) and medulla (m). Right panel shows detail of stromal cells (thymic epithelial cells, TECs; dendritic cells, macrophages, and blood vessels.) Note that mesenchymal cells and the vascular network are omitted for clarity, although the mesenchymal capsule bounding the thymus is shown. Hematopoietic progenitors enter the thymus at the junction between cortex and medulla. Commitment to the T‐cell lineage and differentiation as far as the CD4^+^CD8^+^ ‘double positive’ (DP) stage of development occurs in the cortex. Thymocytes that successfully undergo positive selection can then enter the medulla, which is the site of central tolerance induction. CD4^+^ and CD8^+^ single positive (SP) T cells exit the thymus from the medulla (see 2, 3, 4, 5, 6, 7). DN, CD4^−^CD8^−^ ‘double negative’ thymocytes.

The thymus is a central organ of the adaptive immune system due to its obligatory role in T‐lymphocyte differentiation and repertoire selection 1. These functions depend on the thymic stroma, which comprises a variety of cell types including mesenchymal cells, vascular endothelium, macrophages, dendritic cells and, importantly, a highly specialized epithelial compartment, which confers both structural and functional attributes to the organ. The thymus is divided into two broad regions, the cortex and the medulla (Fig. 1). The epithelial cells in each of these compartments are functionally distinct, with cortical and medullary thymic epithelial cells (cTECs and mTECs, respectively) mediating different aspects of T‐cell development 2, 3, 4, 5, 6, 7. T‐cell development has been reviewed extensively elsewhere (see references 2, 3, 4, 5, 6, 7) and is not revisited in detail herein. Briefly, haematopoietic progenitors enter the thymus at the junction between cortex and medulla. Commitment to the T‐cell lineage and differentiation as far as the CD4^+^CD8^+^ ‘double positive’ (DP) stage of T‐cell development occurs in the cortex. As discussed in detail below, cTECs are required for commitment of haematopoietic cells to the T‐cell lineage, and also mediate both the β selection and positive selection stages of T‐cell lineage development. Developing T cells (called thymocytes) that successfully undergo positive selection can then enter the medulla, the site of central tolerance induction, with tolerance induction being mediated by both mTECs and thymic dendritic cells (Fig. 1). Not surprisingly, in view of their functional differences, cTECs and mTECs are also phenotypically distinct. These differences are discussed in further detail below but in brief, expression of Bp‐1 (En‐pep, the Ly‐51 antigen) by cTECs, and binding of the lectin *Ulex europeaus* agglutinin 1 (UEA1) by mTECs identifies these TEC sublineages and permits their isolation and subsequent analysis. In the adult thymus, Ly‐51^+^ cTECs and UEA‐1^+^ mTECs each constitute heterogeneous populations that can be subdivided into a number of different subsets based on expression of additional surface markers, including MHC Class II 8, 9, 10, 11, 12.

**Figure 2:**
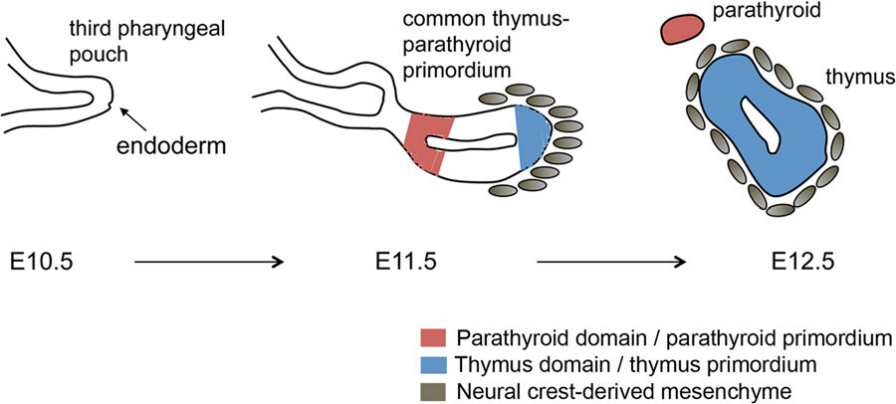
Early events in thymus development. Schematic representation of early thymus development in the mouse. 3PP, third pharyngeal pouch. Gray ovals represent neural crest‐derived mesenchymal cells. Red denotes region of GCM2 expression, marking the parathyroid primordium, blue denotes region of FOXN1 expression, indicating the thymus primordium. E10.5, E11.5, and E12.5 denote day 10.5, 11.5, and 12.5 of embryonic development, respectively.

The epithelial component of the thymus arises from the endoderm of the pharyngeal pouches (PPs). These structures are bilateral outpocketings of the foregut endoderm. The number of PPs varies between species; in mouse and human it is the third PPs (3PPs) that generate the thymus, while other PPs also contribute in some species 13, 14. In mice, the 3PPs form at around day 9 of embryonic development (E9.0). This initial budding is followed by outgrowth and patterning stages, such that each 3PP forms a shared primordium for two organs–the thymus and the parathyroid glands. These organ primordia can be distinguished on the basis of marker expression by E10.5 in mouse, when transcription factor Glial cells missing 2 (Gcm2) mRNA specifically delineates the parathyroid domain, and eventually separate from the pharyngeal endoderm and resolve into discrete organ primordia by about E12.5 15. In humans, the thymus domain within the 3PP is evident by week 6 of gestation 16. The endodermal thymic rudiment within the 3PP is sufficient to direct thymus development, even after transplantation to an ectopic site 17, and appears to contain bipotent thymic epithelial progenitor cells (TEPC) that can generate both cortical and medullary TECs 18, 19, 20, 21. However, the normal process of thymus organogenesis involves interplay between a number of different cell types–including 3PP endoderm, neural crest‐derived mesenchyme, endothelial progenitors, and hematopoietic progenitors–all of which are components of the mature organ (reviewed in 22, 23, 24, 25, 26) (Fig. 2).

Some of the earliest insights into the function of the thymus came from studies on *nude* (*nu/nu*) mice, which carry an autosomal recessive mutation leading to congenital hairlessness and athymia 27, 28. *Nu/nu* mice are correspondingly immunocompromised as they lack normal T‐cell populations 27, 28. The functional athymia in *nu/nu* mice results from a severe developmental block early in thymus organogenesis. The common thymus‐parathyroid primordium forms normally and thymus organogenesis proceeds until E11.5–E12.0. However, a maturational arrest in thymic epithelial progenitor cells occurs at around E12.0 29, such that the *nude* thymic epithelium never becomes competent to support T‐cell development. Indeed, the *nude* thymic rudiment is never colonized by hematopoietic or vascular progenitors; instead, these remain in the perithymic mesenchyme 30, 31. Adult *nu/nu* mice retain a small, cystic, alymphoid thymic rudiment, which does not support T‐cell development at any stage in ontogeny.

## Identification of *Foxn1* as the nude gene

*Foxn1* was originally identified as the gene mutated in *nu/nu* mice using genetic approaches 32, 33. Following localization of *nu* to chromosome 11 in mice and subsequent fine‐mapping, a member of the forkhead or winged helix superfamily, originally named winged helix nude (*Whn;* later renamed *Foxn1*), was identified as the *nude* gene by positional cloning 32, 34. Initial studies showed the *Whn* transcript in *nu/nu* mice carried a single base pair deletion in its third exon, resulting in the absence of *Whn* mRNA due to nonsense‐mediated decay 32. RT‐PCR analysis revealed that *Whn* was expressed in the developing mouse embryo from E9.5, and was restricted to skin and thymus in adult tissues 32. Subsequently, a targeted null allele of *Whn* was generated by inserting a *lacZ M2‐neo* cassette into exon 3 of *Whn*, close to the site of the spontaneous mutation in *nu*. Mice homozygous for this allele phenocopied *nu/nu* mice, confirming *Whn* as the *nude* gene 33.

## The Forkhead family of transcription regulators

FOXN1 was one of the first members of the forkhead (FOX) superfamily of transcription factors (TFs) to be implicated in a specific developmental defect in vertebrates 35. It is now known that this large family of TFs has important roles in the development, homeostasis, function, and aging of a variety of organs and tissues–including the immune system 36, 37. As examples, FOXP3 is needed for the development and function of regulatory T cells (Treg cells) 38, 39, 40, FOXJ1 for suppression of T‐cell activation 41, and FOXO3 for lymphocyte proliferation and apoptosis 42, 43, while FOXN1 itself is essential for production and maintenance of a functional thymus and is also required for hair production 29, 33, 44, 45, 46, 47, 48.

The FOX family is evolutionarily ancient. Its canonical member is the *Drosophila melanogaster* gene *fork head* (*fkh*) which, when mutated, causes a spiked head phenotype in adult flies 49. Identification of the rat gene hepatocyte nuclear factor 3 alpha (*HNF3α*) in 1990 revealed an approximately 100 amino acid region of high homology between the HNF3α and fkh proteins, that was suggested to be a DNA binding domain (DBD) 35, 50. TFs containing this ‘winged helix’ or ‘forkhead’ DBD (Structural Classification of Proteins (SCOP) classification number 46785) were subsequently identified in Eubacteria, Archaea, and Eukaryota, and classified as a new superfamily. More than 2000 FOX family members have now been identified in 108 species of animals and fungi, with numbers differing between species 36, 51, 52, 53. FOX proteins are currently classified based on phylogenetic analysis of their DBDs (forkhead domains), which are highly conserved and represent the only regions of peptide sequence that can be confidently aligned across all FOX proteins 34, 54. Nineteen subclasses of FOX protein have now been identified–FOXA to FOXS–and of these, FOXQ, FOXR, and FOXS are vertebrate‐specific 54.

## The FOXN subfamily

The FOXN genes cluster separately from other FOX subclasses, and the gene most closely related to *FOXN1* is its paralog *FOXN4*. The evolutionary history of the FOXN subclass as currently understood is depicted in Table 1 55, 56. *Foxn4* first appeared in cephalochordates (amphioxus) and is found in all higher organisms. Cephalochordates also contain a more ancient paralog *Foxn4b*, which is present in Echinodermata and Cnidaria but absent from urochordates and all vertebrates. Jawless fish possess a gene very similar to *Foxn4*, termed *Foxn4‐like* (*Foxn4L*). *Foxn1* is thought to be an ortholog of *Foxn4L*, based on protein sequence and short‐range synteny relationships. The expression patterns of these genes further support this genealogy: *Foxn4* (or *Foxn4a*) being expressed in the pharyngeal endoderm and other sites in amphioxus; *FOXN4L*, in the epithelium lining the gill basket in lamprey; and *Foxn1* in the pharyngeal pouches giving rise to the thymus in cartilaginous fishes and all other jawed vertebrates 48. The expression of *Foxn4* in the pharyngeal endoderm in amphioxus suggests that this gene contributed to the evolutionary emergence of thymopoiesis 48. Indeed in evolution, the emergence of FOXN4L and thymus‐like function preceded the pinching off of pharyngeal pouches into a distinct organ, as observed in the lamprey gill basket. Both *Foxn1* and *Foxn4* are expressed in the thymi of catshark, zebrafish, and medaka; however *Foxn4* is either not expressed or expressed at very low levels in higher order organisms 57. Indeed, *Foxn1* appears to have a unique role in the thymus in jawed vertebrates, which cannot be completely substituted by *Foxn4* 57.

**Table 1:** *Foxn1* and its orthologs and paralogs through evolution (adapted from 46)

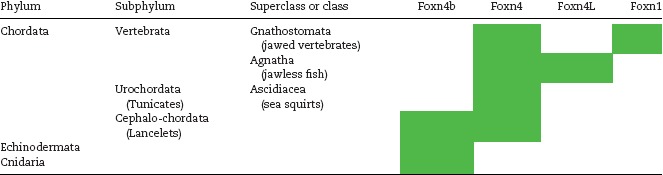

Table indicates the presence or absence of *Foxn1, Foxn4, Foxn4b, and Foxn4l* in different subphyla or superclasses. Based on sequence and synteny homology, *Foxn1* and *Foxn4* are thought to have arisen from a common ancestor gene, *Foxn4b*. Green denotes the presence of the gene in the genome.

## Transcriptional regulation by FOX proteins

The classical ‘forkhead’ DBD consists of three N‐terminal α‐helices (H1, H2, H3), three β‐sheets (S1, S2, S3), and two C‐terminal ‘wing’ regions/loops (W1, W2), arranged in the order H1‐S1‐H2‐H3‐S2‐W1‐S3‐W2 58; an additional α‐helix is present in some FOX proteins 59, 60. The term ‘winged helix’ was coined to reflect the butterfly‐like winged structure adopted by DNA‐bound FOX proteins 59, which resembles structures formed during DNA interactions with linker histones such as H1 and H5 59. The DNA binding specificity of the forkhead domain depends on the variable region at the junction of the α‐helices and wing loops, which interact with bases in minor groove of DNA 61. While all FOX proteins share the forkhead domain, their specific functions are thought to reside in their transactivation or repression domains, which show almost no sequence homology between superfamily members 37. Functional diversity is also determined by differences in interaction partners and spatio‐temporal expression patterns such that, while FOX superfamily members have largely distinct functions, some functional overlap exists between members of the same subgroup 54.

Most FOX factors appear to bind to DNA as monomers 59, however cases of homodimers 62 and heterodimers 63 have also been documented. FOX proteins also interact with nontranscription factor proteins such as co‐activators, co‐repressors, and enzymes. Some FOX proteins also have post‐translational modifications—including phosphorylation, acetylation, methylation, and ubiquitination—which affect their binding affinity and specificity, nuclear localization, and stability 36. Finally, FOX proteins act as effector molecules for several signaling pathways, coupling extra‐cellular signals to changes in gene expression 36.

Recently, much interest has focused on the capacity of some TFs to act at the level of chromatin organization, opening regions of previously compacted chromatin thus enabling their transcription. TFs with this activity are called ‘pioneers’. Pioneer factors are defined as being able to access their target sequence on nucleosomes and certain forms of compacted chromatin, to bind nucleosomes stably and before the binding of other TFs or initiation of the target gene expression, and to possess chromatin‐opening capabilities 64. Forkhead TFs–‐specifically the FOXA proteins FOXA1 and FOXA2 required for hepatic development 65—were among the first to be identified as having ‘pioneer’ function. Detailed investigation of the interaction of the FOXA proteins with chromatin showed they could stably bind their target sequences on in vitro assembled nucleosomes, and could open the local nucleosomal domain through the activity of their C‐terminal domain 66, 67. The similarities between the ‘winged‐helix’ structure of the forkhead domain and structures of linker histones are thought to explain how these TFs can displace linker histones from compacted chromatin, even in the absence of the SWI‐SNF chromatin remodeling complex. Whether FOXN1 has a similar pioneer activity within the TECs remains to be determined. Such activity could explain the broad range of functions regulated by FOXN1 in TECs (see below). However, while the FOXA factors are required for liver specification, FOXN1 does not appear to be required for specification of the TEC lineage (see below), indicating the potential for different functional requirements from these two classes of FOX proteins.

## FOXN1 in thymus development

### Specification of the TEC lineage is independent of FOXN1

As discussed above, low‐level *Foxn1* expression is evident in the pharyngeal endoderm as early as mouse E9.5—the time of the initial outpocketing of the 3PP 32. However, high‐level expression is evident only from E11.25 15. This strong expression initiates in the most ventral tip of the 3PP and subsequently expands to encompass the entire thymus domain (see Fig. 2). Histological analysis has established that FOXN1 is not required for formation of the 3PP or the thymic primordium itself 30, 68 and in keeping with this, several lines of evidence indicate that FOXN1 does not specify the thymic epithelial lineage 17, 29, 48, 68. Following ectopic transplantation, the E9.0 3PPs (which do not yet express *Foxn1*) can generate an intact and functional thymus containing both cortical and medullary thymic epithelial compartments, indicating that 3PP cells are already committed to the TEC lineage 17. Furthermore, both forkhead transcription factor g1 (*Foxg1*) and interleukin 7 (*Il7*) specifically mark the thymus domain of the 3PP and for each, this expression occurs independently of FOXN1 69, 70. Additionally, analyses of revertible null or severely hypomorphic alleles of *Foxn1* have shown that TEPC that lack *Foxn1* expression undergo a developmental arrest that can be reversed in neonatal and adult mice 21, 29, 71. Thus, the fetal TEPC state appears to be extremely stable in vivo, strongly suggesting the presence of a stable transcriptional network upstream of *Foxn1* that confers TEPC identity and thus thymic epithelial lineage specification. Overall, these studies indicate that TEC lineage commitment does not depend solely on FOXN1, implicating an as yet unidentified genetic network in this process.

### FOXN1 in TEC differentiation

As discussed above, the absence of functional FOXN1 arrests fetal TEPC in a bipotent progenitor cell state, and normal functioning in these developmentally arrested fetal TEPCs can be restored by permitting normal FOXN1 expression. This was initially demonstrated using a revertible *Foxn1* allele, *Foxn1* ^SA2^ 21. Using a Cre‐ERT2 system that exhibited low‐level activity in the absence of tamoxifen induction, reactivation of *Foxn1* in a single cell in the thymic rudiment of *Foxn1* null mice was shown to result in the generation of miniature thymi, each containing well‐defined cortical and medullary areas 21. How these findings relate to in vivo development is however still open to question, since *Foxn1* ^−/−^ thymi contain cytokeratin 5^hi^, claudin 4^hi^ (K5^hi^Cldn4^hi^), and K5^−^Cldn4^lo/−^ regions. Since mTEC‐restricted progenitors in the early fetal thymus are Cldn4^hi^ 72, 73, this suggests that the emergence of the mTEC sublineage may be *Foxn1*‐independent and thus that the divergence of the cTEC and mTEC sublineages may occur earlier than implied by this particular genetic analysis 21, 48. Current understanding is therefore that thymus organogenesis can be considered as two stages: early *Foxn1*‐independent development which results in generation of the undifferentiated thymic primordium containing specified TEPCs, and later *Foxn1*‐dependent development in which FOXN1 expression in TEPCs results in their differentiation and the concomitant orchestrated development of the fully patterned and functional thymus 17, 21, 33, 70, 71.

How does FOXN1 effect these later stages of development, and what are its subsequent roles in thymus maintenance and function? Studies on FOXN1 function in early thymus development have revealed a role in TEC proliferation 31 and have also demonstrated its essential role in conferring competence to attract hematopoietic and endothelial progenitors upon TECs in the thymic rudiment 31, 74, 75, 76. Furthermore, FOXN1 has been shown to regulate the maturation and migration of the neural crest cells that will form the thymic mesenchyme 48. Interestingly, the expression of *Vegf‐a* and *Pdgf‐b* in TECs, thymic vasculature‐associated mesenchyme, and endothelium, was severely reduced under conditions of low *Foxn1* expression, with the thymic rudiment showing fewer capillaries, leaky blood vessels, disrupted endothelium–perivascular cell interactions, endothelial cell vacuolization, and an overall failure of vascular organization at later stages of organ development 76, 77. Thus, FOXN1 appears to regulate not only the initial colonization of the thymus with endothelial progenitors, but also normal vascularization of the organ 77.

Further insight into the role of *Foxn1* in TEC differentiation has been provided by studies in which *Foxn1* is either under‐ or overexpressed specifically in TECs. For example, mice homozygous for a hypomorphic *Foxn1* allele, *Foxn1* ^Δ^, whose transcript lacks the N‐terminal domain of FOXN1, develop severely hypoplastic and cystic thymi that lack distinct cortical and medullary regions 44. T‐cell development appears relatively normal in the fetal *Foxn1* ^Δ/Δ^ thymus. However, the adult *Foxn1* ^Δ/Δ^ thymus supports only aberrant thymopoeisis, characterized by the absence of CD25^+^ thymocytes (DN3 cells), and reduced expression of TCR‐β at the DP stage. Analysis of *Foxn1* ^Δ/Δ^ mice is consistent with TEC differentiation being initiated, but then blocked at an intermediate stage of development, such that the *Foxn1* ^Δ/Δ^ thymus has impaired functionality compared with that of the wild‐type 44. A second severely hypomorphic *Foxn1* allele, *Foxn1^R^*, allowed investigation of FOXN1 function in TEC differentiation independently of its role in proliferation 48. *Foxn1* ^R^ generates only around 15% of WT levels of normal *Foxn1* transcripts, which results in development in *Foxn1^R/R^* mice of a hypoplastic thymus which can only suboptimally support T‐cell development such that fewer thymocytes are generated during differentiation. Analysis of an allelic series based on the *Foxn1^R^, Foxn1^−^*, and wild‐type *Foxn1* alleles revealed strong dose‐dependent effects of *Foxn1*, in brief showing that increasing levels of *Foxn1* expression are required for progression through multiple intermediate stages of TEC development—from exit from the earliest progenitor cell state(s) through to terminal differentiation, in both cTEC and mTEC sub‐lineages in the fetal and adult thymus 48.

Despite 20 years having elapsed since confirmation of its identity as the *nude* gene product, the molecular functions of FOXN1 have not yet been determined in full, and indeed no direct targets have yet been verified in TECs by chromatin immunoprecipitation. However, FOXN1 has been shown to be required for the expression in TECs of proteins with essential roles in promoting thymocyte development, including Chemokine (C‐C Motif) Ligand 25 (CCL25), C‐X‐C motif chemokine 12 (CXCL12; also known as stromal cell‐derived factor 1 (SDF1), Delta‐like ligand 4 (DLL4), Stem cell factor (SCF; also known as SCF, KIT‐ligand, KL, or steel factor), Cathepsin L (CTSL), the 20S proteasome subunit beta‐5t (β5t; also known as Psmb11), and MHC Class II 45, 48, 78. CCL25 and CXCL12 are chemokines which are required for attracting thymic seeding cells into the developing thymic rudiment and the adult thymus 74, 79, 80. DLL4 is a Notch ligand required for TECs for commitment of hematopoietic progenitors to the T‐cell lineage 81, 82, and SCF is required for thymocyte survival and proliferation 83. Cathepsin L and β5t regulate the production of peptides required in TECs to effect optimal positive selection of CD4^+^ and of CD8^+^ thymocytes, respectively 84, 85, 86, 87, while MHC Class II expression is critical for positive and negative selection of CD4^+^ T cells. Notably, transgenic expression of *Dll4*, *Ccl25*, *Cxcl12*, and *Scf* conferred some capacity to support production of CD4^+^ and CD8^+^ SP T cells to the *Foxn1* ^−/−^ thymic rudiment, although the TEPCs within the rudiment remained in an undifferentiated state 78. Since the *Foxn1* ^−/−^ thymus expressing transgenic *Dll4*, *Ccl25*, *Cxcl12*, and *Scf* lacked functional TECs and normal thymus architecture, this study established that *Foxn1* must regulate additional genes that are required for TEC differentiation and function. In keeping with this observation, in addition to the genes discussed above, the genes encoding transformation related protein 63 (*Trp63*), Paired Box 1 (*Pax1*), Fibroblast growth factor receptor 2 isoform IIIB (*Fgfr2IIIb)*, Autoimmune regulator (*Aire*), Cluster of differentiation 40 (*CD40*), Cluster of differentiation 80 (*CD80*), and Cyclin D1 (*Ccnd1)*, which have known roles in TEC differentiation, proliferation, or function, and of several genes involved in Wnt signaling, are all FOXN1 responsive in TECs 48, 88. Thus, although further insights, including delineation of which of these genes are direct FOXN1 targets, are undoubtedly required, the range of genes and breadth of functions known to be affected by FOXN1 expression already provides an indication of how this single TF can orchestrate thymus organogenesis and function.

### *Foxn1* in thymus homeostasis and involution

TECs in the adult thymus continue to express *Foxn1* 33, with cTECs expressing higher levels than mTECs, and MHC Class II^hi^ cells expressing higher levels of *Foxn1* than MHC Class II^lo^ TECs in each compartment 48, 89, 90, 91. Several studies have demonstrated the importance of FOXN1 in maintenance of the adult thymus 45, 46, 47. Interestingly, down‐regulation of *Foxn1* expression in the thymic stroma is one of the earliest events in the age‐associated degeneration of the thymus 92, suggesting that FOXN1 could play an important role in postnatal thymus homeostasis and subsequent thymic involution. This hypothesis was supported by analysis of a *Foxn1* allele (*Foxn1* ^lacZ^), which expresses normal levels of *Foxn1* in the fetal and newborn thymus, after which *Foxn1* expression declines to 20–30% of wild‐type levels by 5 weeks after birth 45. *Foxn1* ^lacZ/LacZ^ mice exhibit a premature loss of thymus homeostasis, correlating with *Foxn1* downregulation, that phenocopies many of the hallmarks of age‐related involution 45. The TEC subsets most affected were those that normally express high levels of *Foxn1*, indicating their continued FOXN1 dosage sensitivity 45. This study provided the first functional evidence linking FOXN1 down‐regulation with age‐related thymic involution. Consistent with this, ubiquitous deletion of *Foxn1* or of FOXN1^+^ cells in postnatal mice, resulted in rapid thymic atrophy, further supporting a role for FOXN1 in thymus homeostasis 46, 47, 93.

*Foxn1* expression in the involuting thymus has recently been investigated at the single cell level. Using a new antibody generated against the C‐terminus of FOXN1 protein and a tagged version of FOXN1, Rode and colleagues showed that in aging mice, *Foxn1* expression progressively decreases and there is an age‐related accumulation of *Foxn1* ^−/low^ TECs 90. Lineage tracing of *Foxn1*‐negative TECs has shown that these cells arise from Foxn1‐positive precursors 47, 91, 94. A second study used a *Foxn1*‐eGFP reporter mouse line (in which eGFP was knocked into the *Foxn1* locus) to investigate transcriptional changes in *Foxn1* expression with age, and similarly showed that the emergence of *Foxn1* ^−^ TECs correlates with the onset of age‐related thymic involution 91. This study suggested down‐regulation of FOXN1 in a subset of cTECs as a primary event in age‐related thymic involution, and further showed that this FOXN1 downregulation correlated with diminished cTEC functionality based on decreased expression of genes required in cTECs to promote T‐cell differentiation. Together, these analyses suggest that both the onset and progression of involution are the result of declining *Foxn1* expression.

**Figure 3:**
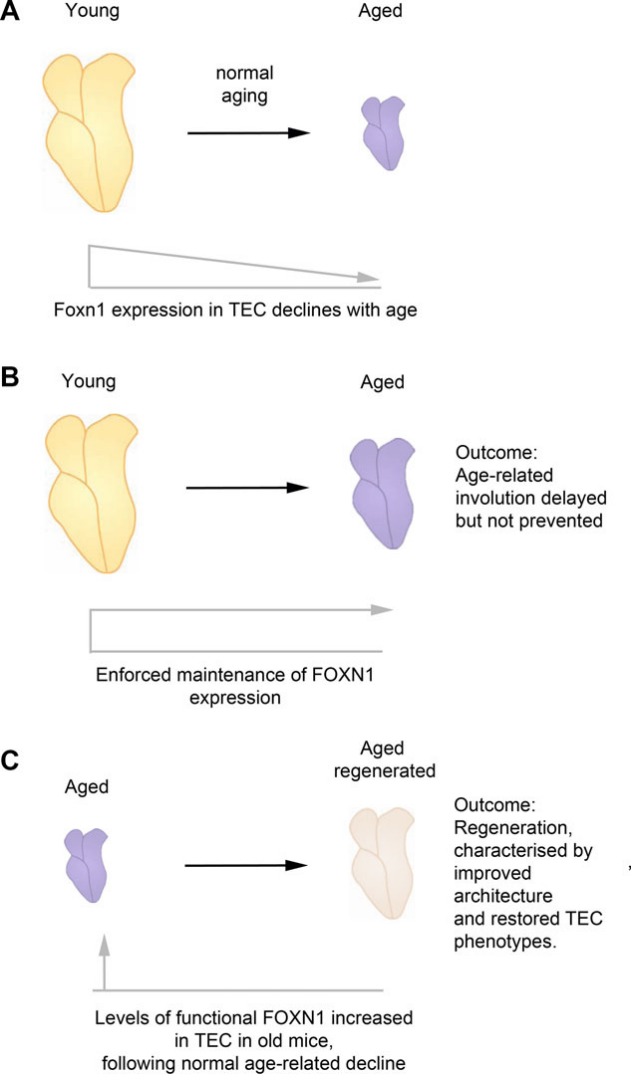
Foxn1 expression levels and thymic involution. The level of expression of *Foxn1* is correlated with and influences age‐related thymic involution. (A) During normal healthy aging, FOXN1 expression levels decrease concomitant with the decrease in size, organization, and TEC functionality that characterizes age‐related thymic involution. (B) Enforced high‐level FOXN1 expression in TECs, as with the *Foxn1*tg transgene, delays but does not prevent age‐related involution. (C) Induction of increased levels of functional FOXN1 in TECs in the fully involuted, aged thymus, leads to true thymus regeneration evidenced by increased thymus size, increased thymopoiesis and output of naïve T cells, and restoration of thymus architecture and TEC phenotype to close to those of the young thymus.

The emerging evidence, discussed above, suggesting that down‐regulation of FOXN1 might be a primary cause of age‐related thymic involution has recently been tested in two studies, which have respectively determined the outcome of maintaining high‐level FOXN1 expression throughout ontogeny 95, and of up‐regulating FOXN1 function in the aged thymus 88. The first used a strain of transgenic mice, hK14‐Foxn1 (also known as Foxn1tg). In this strain the mouse *Foxn1* cDNA, under control of the human K14 promoter, was introduced into the genome by random insertion, resulting in 20‐fold over‐expression of *Foxn1* in TECs due to multiple copies of the transgene 95. These mice initially exhibited increased thymus size, with increased thymic output and numbers of early thymocyte progenitors (ETPs) 95. However, although age‐related thymic involution was delayed in this model, it was not prevented (Fig. 3A and B) 95. This suggested that FOXN1 is a target in age‐related thymic involution, but that other targets might also exist.

**Figure 4:**
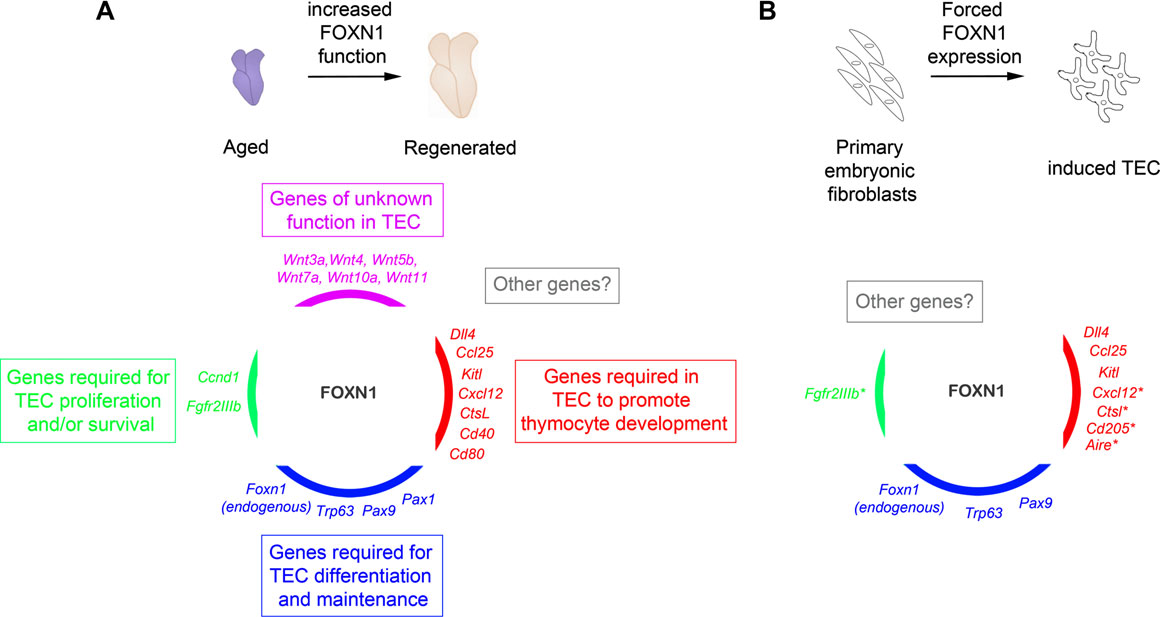
FOXN1–a master transcriptional regulator of TEC gene expression. FOXN1 directly or indirectly regulates the expression of a number of genes in TECs during *Foxn1*‐induced thymus regeneration (A) and transdifferentiation of MEFs to iTECs (B). The genes regulated by FOXN1 are responsible for a variety of functions in TECs, including differentiation, maintenance, and function, as well as including those with as yet unknown functions. Lower panel in (A) shows genes whose expression in TECs is downregulated with age, and restored to close to juvenile expression levels when FOXN1 function is increased in aged TECs, grouped according to their known functions in TECs. Lower panel in (B), TEC identity and function is evidenced by expression of the genes shown. * denotes genes whose expression was demonstrated in MEF‐derived iTECs recovered after transplantation but which were not present or not tested in iTECs prior to transplantation. Note that the cohort of genes regulated by FOXN1 in (A) and (B) is likely to be broader than depicted here.

The second study, from this laboratory, used a novel transgenic mouse strain, R26‐*Foxn1ERT2*, which allows tissue‐specific expression of a tamoxifen‐regulatable form of FOXN1 (FOXN1ER) 88. Using this model, we showed that increasing FOXN1 activity in TECs in 12‐ or 24‐ month old mice resulted in thymus regeneration, characterized by restoration of thymic architecture and functionality close to that found in young mice 88 (Fig. 3C). This up‐regulation of FOXN1 function led to increased expression of genes important for TEC biology and function, including *Dll4*, *Ccl25*, *Kitl*, *Cxcl12, Ctsl, Cd40, Cd80, Pax1*, *Trp63*, *Fgfr2IIIb*, and *Aire*, to levels similar to those observed in the young thymus (Fig. 4A) 88. It also led to increased proliferation in immature TEC subsets, strongly suggesting that the observed thymus regeneration was instigated by a coordinated proliferation and differentiation of TEC progenitors 88. These data show that up‐regulation of FOXN1 function is sufficient to drive regeneration of the aged thymus, establishing FOXN1 as the primary target of the mechanisms driving age‐related thymic involution. Of note is that uncontrolled differentiation of TEC progenitor/stem cells was not indicated in either the K14‐Foxn1 or R26‐Foxn1ERT2 models 76, 82, suggesting that other factors must interact with FOXN1 to regulate the balance between proliferation and differentiation.

### FOXN1: A master transcriptional regulator of TECs

The evidence discussed above has established FOXN1 as a powerful mediator of TEC differentiation and maintenance. Remarkably, recent work from this laboratory has shown that overexpression of FOXN1 in an unrelated cell‐type, mouse embryonic fibroblasts (MEFs), is sufficient to reprogram the MEFs into functional TECs 96 (Fig. 3B). These FOXN1‐induced MEFs were shown to express genes indicative of TEC lineage identity, including *Dll4*, *Ccl25*, and *Kitl* (Fig. 3B), and to provide a permissive environment for the maturation of ETPs to DP and SP thymocytes in vitro 96. Furthermore, these ‘induced’ TECs (iTECs), upon transplantation under the kidney capsule of 5–6 week old *nu/nu* or syngeneic wild‐type mice together with supporting thymic mesenchymal cells and immature thymocytes, went on to generate a fully functional thymus, with characteristic cortical and medullary architecture 96. The iTECs were shown to express endogenous *Foxn1*, consistent with the positive auto‐regulation of *Foxn1* observed in the *K14‐Foxn1* transgenic mice, and iTECs recovered after transplantation expressed a range of genes required for TEC differentiation, proliferation, and function (Fig. 4B) 95, 96. This study thus extends previous understanding to establish that FOXN1 functions as a master regulator of TEC differentiation, which is capable of initiating and maintaining the transcription factor network required to promote TEC identity (Fig. 4).

### Regulation of *Foxn1* expression in TECs

Given the importance of FOXN1 in thymus biology, there has been considerable interest in its upstream regulation. However, information regarding transcriptional regulators of *Foxn1* is surprisingly scarce. Concrete evidence supports positive autoregulation of *Foxn1* in TECs 95, 96. However, whether this is direct or indirect remains to be determined. Recently, some members of the E2F family of transcription factors (E2Fs; specifically E2F3 and E2F4), which mediate cell cycle progression among other functions and are negatively regulated by Retinoblastoma tumor suppressor (Rb) proteins, have been shown to be able to bind to their consensus binding site in the presumptive *Foxn1* promoter in vitro 97. Additionally, increased activity of E2F3 in vivo was shown to correlate with increased expression of *Foxn1* in TECs 97. This link between E2F3 activity and *Foxn1* expression was revealed by analysis of compound transgenic mice that lack the Rb family genes Rb and p103, and carry only a single copy of the third Rb family member p107 (Mx1‐Cre;Rb^lox/lox;^ p130^lox/lox;^ p107^+/−^: called *Mx1‐Cre p107‐single* mice). By 9 months old, these mice exhibit severe thymus hyperplasia, characterized by increased cellularity and increased *Foxn1* expression levels in TECs. Further genetic analysis showed that reduced levels of *Foxn1* expression (achieved by breeding the *Foxn1^LacZ^* allele onto the Mx1‐Cre p107‐single background) were sufficient to reverse this hyperplastic phenotype, implicating RB and hence E2Fs in *Foxn1* regulation 97. Another candidate transcriptional regulator of *Foxn1* in TECs is the T box transcription factor TBX1, mutation of which is thought to cause DiGeorge Syndrome 98, 99: induced expression of TBX1 in *Foxn1* expressing cells of the E11.5 3PP resulted in down‐regulation of *Foxn1* expression, indicating that TBX1 can repress *Foxn1* transcription in TECs 100. Consistent with its expected effect on *Foxn1* repression, the forced TBX1 expression in *Foxn1* ^Cre^;*R26‐iTbx1* thymi appeared to block TEC differentiation in an early progenitor state, evidenced by the accumulation of progenitor‐phenotype cells (characterized by expression of Placenta expressed transcript 1; PLET1) and the absence of differentiated TECs in the fetal thymus 100. However, it remains to be determined whether TBX1 regulates *Foxn1* directly or indirectly, and in this light TBX binding sites have not yet been identified in the putative *Foxn1* promoter regions 100. Transcriptional regulation of *Foxn1* in the hair follicle and skin may also provide some clues as to its regulation in TECs, and notably, a homeobox family member, *Hoxc13*, has been suggested to regulate *Foxn1* in skin and hair follicle. However whether *Hoxc13* is also involved in regulation of *Foxn1* expression in TECs remains to be determined 101. Finally, the BMP and WNT‐signaling pathways have been implicated in regulating *Foxn1* expression in TECs 102, 103, 104, 105, 106, 107, 108, 109, 110, 111, although again, the molecular details have not been reported.

Surprisingly, the regulatory regions governing *Foxn1* expression in TEPCs or TECs are also still only poorly characterized. Several studies have investigated whether a minimal genomic region surrounding the *Foxn1* gene on mouse chromosome 11 can reproduce the wild‐type *Foxn1* expression pattern in skin and thymus. The largest region tested was 110kb, containing the entire *Foxn1* locus with an additional 74kb of 5′‐flanking sequence and 12kb of 3′‐flanking sequence; this region rescued the *nude* phenotype in vivo, indicating that it contains all the regulatory elements required for normal expression of *Foxn1* 112. A 26kb region of genomic DNA encompassing the coding exons of *Foxn1* plus 8.5kb of 5′‐flanking sequence and 3kb of 3′‐flanking sequence could rescue the hairless but not the athymic phenotype of *nude* mice, showing that it lacked at least some of the regulatory regions required for *Foxn1* expression in TECs 113. However, a 30kb fragment containing the entire upstream sequence between the first coding exon of *Foxn1* (exon‐2) and the upstream gene *Slc13a2* can recapitulate *Foxn1* expression pattern in the developing thymus 114, although definitive characterization of its capacity to drive normal *Foxn1* expression in the postnatal and adult thymus has not been provided. Within these regions, the promoter and enhancers governing the expression of *Foxn1* in TEPCs and TECs remain to be definitely identified and similarly the identity of the tissue‐restricted transcription factors important for its expression remains elusive.

## Conclusion

FOXN1 plays a critical role in thymus biology, functioning as a master regulator of TEC differentiation, function, and maintenance in the fetal and adult thymus and displaying remarkable potency as a regeneration and reprogramming factor. Further investigation of FOXN1 function will thus illuminate TEC biology during development, homeostasis and aging, and contribute to a broader understanding of how master regulator TFs function to regulate and coordinate gene expression programs. Elucidation of the transcription factor networks responsible for regulating the initiation and maintenance of *Foxn1* in different TEC subsets will also be crucial. This presents a major challenge, but should now become tractable in light of recent technological advances allowing interrogation of global gene expression and TF binding in single cells/small cell populations. In this regard, the recent identification of TBX1 and E2F as transcriptional regulators of *Foxn1* should provide a tangible starting point for deciphering the molecular details of *Foxn1* transcriptional regulation.

## Conflict of interest

C.C.B. and A.B.L. declare no financial or commercial conflict of interest. H.J.V. is a consultant for a privately held company.

**Abbreviations**
cTECs: cortical thymic epithelial cells
DN: double negative
DP: double positive
ETPs: early thymocyte progenitors
FOX: forkhead
mTECs: medullary thymic epithelial cells
MEFs: mouse embryonic fibroblasts
PP: pharyngeal pouch
SP: single positive
TECs: thymic epithelial cells
TEPCs: thymic epithelial progenitor cells
